# Assessing sleep, cognition and overnight memory performance in neurotypically developing youth in a children's hospital

**DOI:** 10.3389/frsle.2026.1771860

**Published:** 2026-06-04

**Authors:** Gabrielle N. Deutsch, Emmet W. Klein, Chelsea E. Cadle, Katharine C. Simon, H. Gerry Taylor, Maninder Kalra, Paola Malerba

**Affiliations:** 1 Center for Biobehavioral Health, The Abigail Wexner Research Institute, Nationwide Children's Hospital, Columbus, OH, United States; 2 Department of Pediatrics, School of Medicine, University of California, Irvine, Irvine, CA, United States; 3 Pulmonology Department, Children's Hospital of Orange County, Orange, CA, United States; 4 The Ohio State University School of Medicine, Columbus, OH, United States; 5 Division of Pulmonary and Sleep Medicine, Nationwide Children's Hospital, Columbus, OH, United States

**Keywords:** children's hospital, neurotypical cognition, polysomnography, sleep, slow oscillations

## Abstract

Childhood and adolescence are times of significant maturation for cognitive abilities and cortical neurophysiology, and changes in sleep brain activity across development reflect these maturation trajectories. Furthermore, sleep is known to support changes in cognition across the lifespan, including in domains of memory, emotion regulation and executive functioning. Driven by our interest in establishing mechanistic links between changes in sleep neurophysiology and cognitive/mood symptoms in pediatric populations, we aim to establish research protocols for examining sleep-dependent cognition in a wide range of pediatric populations. As a first step, we have established a data acquisition protocol that pairs measurement of overnight memory changes with polysomnography involving a full electroencephalography (EEG) montage and standardized clinical assessments of cognition. While this is typical of a cognitive sleep laboratory, we are now executing it in the context of a pediatric hospital. The protocol is essential in creating an inclusive research program where children and adolescents with a range of conditions can participate in research in a context that is safe and tailored to their needs (a sleep center in a pediatric hospital). This article describes our first protocol, developed for typically developing youth aged 8–19 years, including collection of subjective and objective measures of sleep and cognition, emotion regulation and executive functioning, and overnight changes in memory performance. We also share strategies for ensuring data quality in our protocol.

## 1 Introduction

Across childhood and adolescence, age-associated changes in sleep parallel the development of cognitive domains (Galland et al., 2012; Mason et al., 2021; Simon et al., 2025), including memory (Hahn et al., 2019; Lokhandwala and Spencer, 2021; Simon et al., 2022b) and executive functioning (Vermeulen et al., 2019; Morales-Ghinaglia et al., 2025). Maturational changes in cortical connectivity are understood to drive the parallel changes in sleep brain dynamics and cognition. For example the topographical organization of the peak density in slow wave activity (SWA, 0.1–4 Hz) during non-rapid-eye-movement (NREM) sleep is understood to track frontal lobe maturation across development (Kurth et al., 2010). In youth, sleep brain oscillations including slow oscillations (SO, 0.5–1.5 Hz) and spindles (10–16 Hz) have been tied to cognitive and mood outcomes such as IQ, depression, anxiety, and executive functioning (Kopasz et al., 2010; Gruber et al., 2013; Kurdziel et al., 2013; Turnbull et al., 2013; Doucette et al., 2015), with properties of SOs like density and coordination correlated to assessment scores in these cognitive and mood domains. Similarly, overnight changes in performance in declarative and procedural memory tasks in youth have also been linked to SOs and spindles. Examples include associations of increased density of sleep spindles during a nap with increased accuracy after the nap in an episodic memory task in pre-school children (Kurdziel et al., 2013) and coordination between SOs and spindles correlating with recall in a verbal memory task (Kurz et al., 2023).

Research documents sleep disturbances in a number of clinical conditions, including chronic disease (e.g., substance use disorder, chronic pain, diabetes) (Onen et al., 2001; Pillar et al., 2003; Finan et al., 2013; Caruso et al., 2014; Hartwell et al., 2014) mood disorders [e.g., depression (Lovato and Gradisar, 2014; Palmer and Alfano, 2017), anxiety (Johnson et al., 2006), schizophrenia (Afonso et al., 2014)], and genetic disorders (e.g., Down syndrome (Maris et al., 2016; Chawla et al., 2021), muscular dystrophies [Barbe et al., 1994; Sawnani et al., 2015]. Abnormalities in sleep architecture and neurophysiology have been demonstrated in many of these conditions, such as depression (Wichniak et al., 2013), Down syndrome (Ellingson and Peters, 1980; Shetty et al., 2024) Duchenne Muscular Dystrophy (Simon et al., 2021, 2022a), and schizophrenia (Kozhemiako et al., 2023; Ferrarelli, 2024). In fact, recent reviews describe differences between clinical populations and neurotypical controls in sleep neurophysiology (Tamir et al., 2023; O'Regan et al., 2025). Notably, literature documenting associations of alterations in sleep physiology in clinical populations with objectively measured cognitive results remains sparse (Jauch-Chara et al., 2007; Maski et al., 2025; Morales-Ghinaglia et al., 2025).

This article describes a protocol that seeks to further elucidate the complex relationship between sleep, cognition, and mood in neurotypically developing children and adolescents. The protocol includes objective and subjective assessment of sleep, cognition, and mood, including testing of overnight changes in performance in episodic and perceptual memory tests. Assessment involves coordination of sleep polysomnography (PSG) at our Sleep Center, which includes a full montage head electroencephalography (EEG), with administration of cognitive and mood measures at the Center for Biobehavioral Health within our research institute. The primary study aim is to establish age-dependent changes in the space-time organization of sleep slow oscillations on the electrode manifold. The secondary study aim is to explore if metrics that quantify the space-time organization of sleep slow oscillations are related to sleep-dependent changes in episodic memory or perceptual memory in youth.

Of note, we developed this protocol with the intention of also implementing substantially similar protocols with clinical populations in the future. Consistent with this long-term goal, the protocol requires adapting methodologies developed in non-clinical research laboratories for use within a hospital setting that treats children for clinical disorders. A fundamental reason to conduct polysomnography in research on clinical populations is to combine expertise in sleep medicine and sleep assessment with administration of behavioral measures. Staff members are experienced in working with health-related conditions and with methods of data collection and analysis. Clinical polysomnography also allows evaluation of incidental findings and integration of clinical sleep expertise in the study team, which is crucial in study of populations with known sleep issues or that take medications that are known to affect sleep and/or breathing and cardiac activity. Our paradigm also leverages the clinical research infrastructure established at our hospital, where research teams that study biobehavioral health have access to research-dedicated spaces that can host data collection for cognitive assessments.

## 2 Methods and analysis

### 2.1 Measures

**Figure 1:**
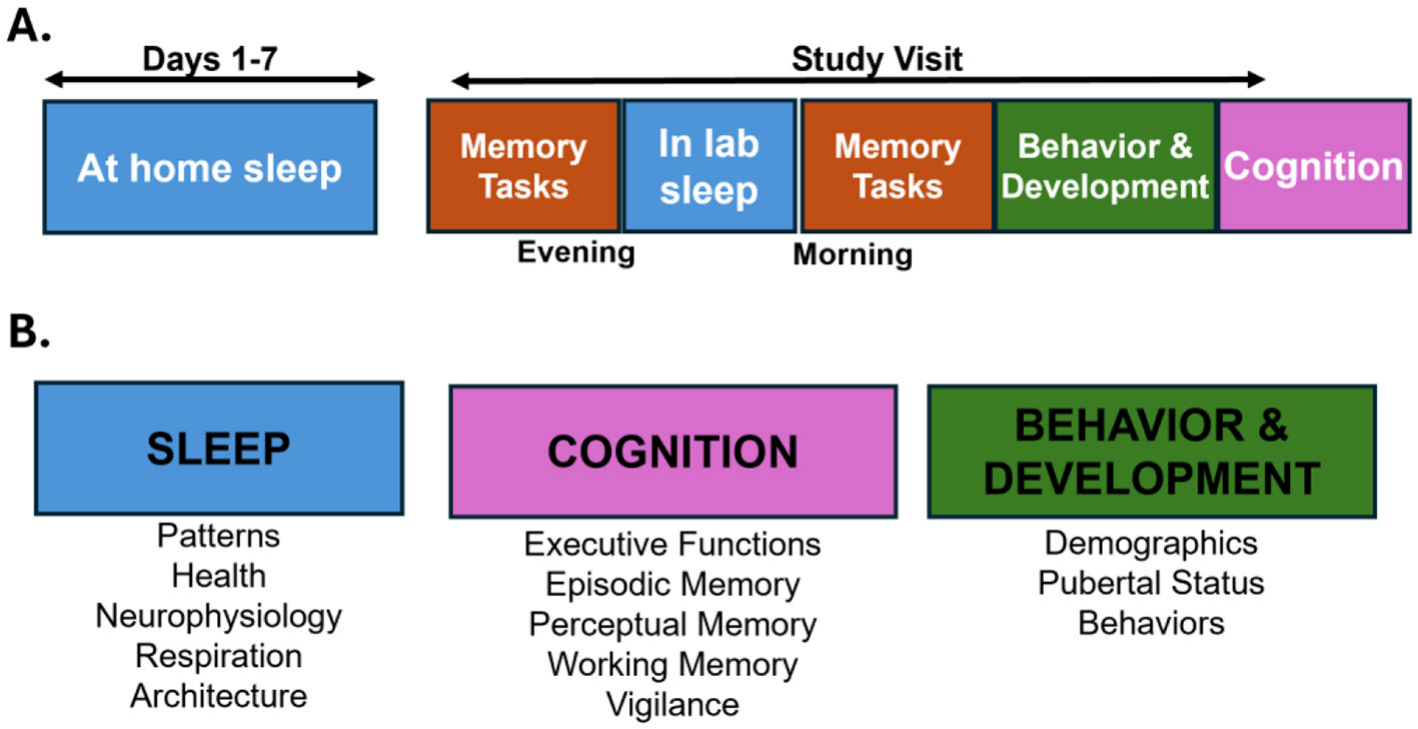
Study visit timeline. **(A)** A representation of the timeline of a study visit in our protocol. **(B)** The domains of development, sleep, cognition, behaviors, and sleep-dependent memory addressed by measures in the protocol.

Our protocol collects data across multiple domains: sleep, cognition, development, and emotion regulation. Below is a detailed description of each measurement domain. We share the rationale we used in choosing each measure in Supplementary Table S1. A visual representation of a study visit timeline and the domains addressed by this protocol is shown in Figure 1.

#### 2.1.1 Demographics

Caregivers/legal guardians are asked to complete a demographic questionnaire (including date of birth, race and ethnicity, gender, presence/absence and type of disability, and socioeconomic status as derived from zip codes). Participants also complete the pubertal development scale (PDS) (Petersen et al., 1988; Koopman-Verhoeff et al., 2020), which is a seven-item self-report survey on physical development including questions on body hair growth, acne, growth in height, etc.

#### 2.1.2 Objective sleep

Child participants complete an overnight PSG, conducted within the hospital Sleep Center. Each acquisition leverages a full EEG montage (24 electrodes 10–20 system by hand), significantly increasing the density of head electrodes in the PSG compared to standard clinical practice. This is necessary to enable high-quality analyses of sleep oscillations and their organization in space-time (Mölle and Born, 2011; Malerba et al., 2022). PSG also leverages nasal cannulas, respiratory belts, leg leads etc. to measure respiration, body movement, eye movement, and heart rate. After acquisition, registered sleep technicians score the PSG according to AASM guidelines (Troester et al., 2023) and a Sleep Medicine physician reviews each sleep study for potential incidental findings. Incidental findings for this study were defined based on AASM ICSD Manual, 3rd edition (American Academy of Sleep Medicine, 2014). If any finding is of potential clinical relevance, the study team will inform the family of the incidental finding, and of their option to receive specifics of the findings either directly from the Sleep Medicine physician on the research team, or to have a family physician contact the sleep medicine expert to discuss those findings.

#### 2.1.3 Self-reports of sleep

We collect information on sleep at home to establish patterns of sleep behaviors for our participants. Youth self-reports include the Epworth Sleepiness Scale for Children and Adolescents (ESS-CHAD) (Johns, 2015; Janssen et al., 2017) for participants up to 18 years of age and younger, and the Epworth Sleepiness Scale (Johns, 1991; Doneh, 2015) for participants 19 of age and older. Participants also complete a 7-day Sleep Diary, which was developed using the National Sleep Foundation Diary (Carney et al., 2012; Knutson et al., 2017) adapted for teenagers. Participants log morning and evening behaviors in their diary to establish at-home sleep across the week. All questions included in the diary are reported in Table 1, with an example printout provided in Supplementary Figure S1. Note that our group keeps track of the actual start date of each sleep diary, allowing for identification of weekdays and weekend days in data collected via sleep diary.

**Table 1:** Sleep diary.

Complete in the morning	Day 1	Day 2	…
I got into bed last night at…			
I fell asleep at…			
I fell asleep…	□ Easily □ After some time □ With difficulty	□ Easily □ After some time □ With difficulty	□ Easily □ After some time □ With difficulty
I woke up during the night…	#of times #of minutes	#of times #of minutes	#of times #of minutes
I woke up this morning at…			
I got out of bed at…			
When I woke up for the day, I felt…	□ Rested □ Somewhat rested □ Tired	□ Rested □ Somewhat rested □ Tired	□ Rested □ Somewhat rested □ Tired
Complete in the evening			
I consumed caffeinated items in the: *(M)orning, (A)fternoon, (E)vening, NA* How much?			
I exercised at least 20 minutes in the: *(M)orning, (A)fternoon, (E)vening, NA*			
I took these medications today:			
Took a nap? Yes or No If yes, for how long?			
During the day how likely were you to nod off or even fall asleep while performing daily tasks: *No chance (NC), Slight chance (SC), Moderate chance (MC), High chance (HC)*			
Throughout the day my mood was… *Very pleasant (VP), Pleasant (P), Unpleasant (U), Very unpleasant (VU)*			
In the hour before going to sleep my bedtime routine included: *List of activities including reading a book, watching tv, exercising, etc*.			
In the hour before going to bed I used electronics: *iPad, phone, computer*			

#### 2.1.4 Cognition

Participants complete memory tasks taken from the cognitive science literature, as well as assessments utilized in clinical practice. Study coordinators administer memory tasks the evening before and the morning after the PSG night.

**Figure 2:**
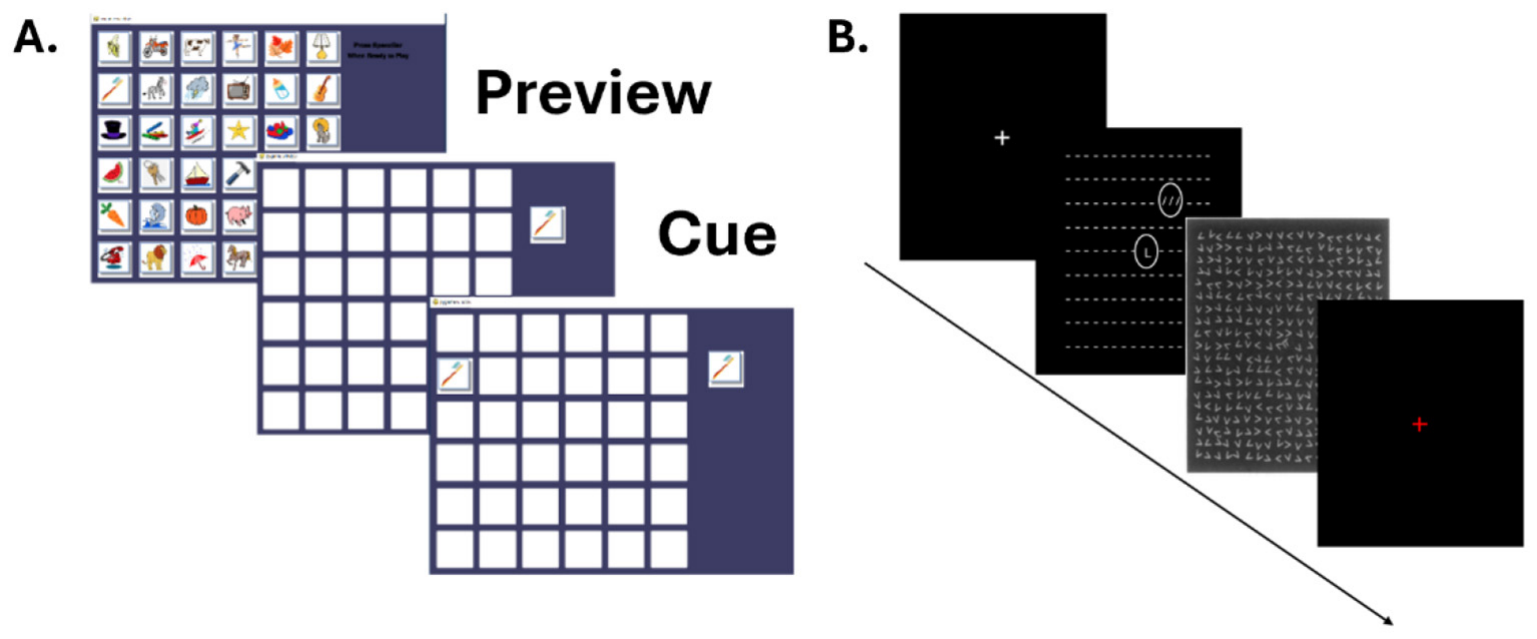
Card grid task and texture discrimination task. **(A)** Representation of the card grid task administration. Participants are first presented the entire grid of pictures and are then prompted to identify the location of each picture. **(B)** Representation of the texture discrimination task administration. Participants are introduced to a stimulus that indicates the start of a trial. In each trial participants are instructed to press number keys that represent a target symbol (circled) and the orientation of diagonal lines in a quadrant.

The Card Grid Task (CGT) is an episodic memory task developed by Kurdziel et al. for preschool children 36–67 months of age (Kurdziel et al., 2013). Previous studies have found that performance in this task benefits from a period of sleep compared to an equivalent time of wake (Diekelmann et al., 2009; Wilhelm et al., 2012). Participants complete the task on a laptop connected to a monitor with a large enough screen to display all elements of the memory task (grid of cards and card cue space) in one comprehensive screen view, which removes the need for use of navigation bars when playing the CGT. During encoding, participants are presented with a grid of pictures (cards) and are asked to name and point to each on the grid (e.g., four pictures placed in a 2 × 2 grid and nine pictures in a 3 × 3 grid, see Figure 2A). After the grid is cleared, each picture is presented at random to the participant, who is asked to point to the position of the picture in the grid. The full set of pictures used in the grid is presented to the participant as a randomized set, and the fraction of pictures that got a correct location is evaluated after the full set is presented. If the participant located correctly fewer than 75% of the pictures, the full set of cards is randomized again and presented to the participant, one at a time. This process continues until the participant can correctly identify the location in the grid of 75% of the pictures. To ensure appropriate challenge level and prevent ceiling learning effects, older children are required to learn the positions of more items in larger grid sizes (up to grids of 6 × 6 for participants 8–11 years of age and 7 × 7 for participants 12–19 years of age). Directly following encoding, participants complete the immediate test (T0) in which the grid is again cleared, and the participant is asked to identify the position of each of the pictures one time, presented one at a time from a randomized full set of cards. This post-training test is then repeated the morning following the PSG (T1).

The Texture Discrimination Task (TDT) is a procedural memory task utilizing discriminatory processing of texture and fixation targets rooted in a patterned background. Performance in this task has been shown to benefit from sleep (Ahmadi et al., 2018; McDevitt et al., 2018; Shuster et al., 2024). When taking the test, participants must make two button presses per trial using a standard keyboard. Each trial consists of a fixation display lasting 13.3 ms, a blank pre-stimulus interval lasting 106.4 ms, the target display lasting 26.6 ms, a blank interstimulus interval (ISI) of varying duration, the mask lasting 13.3 ms, and a second fixation display which cues the participant to respond lasting 2,000 ms (see Figure 2B). The training session of the task includes five blocks presented at ISI of 997.5 864.5, 731.5, 585.2, and 319.2 ms, for a total of 125 trials. Participants are prompted to focus on the middle of the screen indicated by a small cross. They are then asked to select specific keys on the keyboard associated with the target symbol and the orientation of diagonal lines that appear in either the left or right corner (dependent on the randomization tool). They first respond to the target symbol (“1” for L and “2” for T) and then the orientation that is presented using diagonal lines (“1” for vertical orientation and “2” for horizontal orientation). Training continues until participants complete one run with at least 80% accurate discrimination of both the fixation and texture targets. Orientation of the texture target switches across participants and is presented either at the top left or top right corner of the screen which is determined by a randomization tool. The immediate test conducted prior to PSG (T0), consists of 13 blocks, or 325 total trials, with ISI intervals of 598.5, 505.4, 399, 305.9, 252.7, 199.5, 159.6,146.3, 119.7, 106.4, 66.5, 26.6, and 0 ms. To assess sleep dependency, a delayed session (T1) occurs the morning after PSG.

The Psychomotor Vigilance Task NASA PVT+ (PVT) (Basner and Dinges, 2012), is completed directly following awakening from PSG as an indicator of restfulness. Our study utilizes the 5-min adaptation of this task administered on an iPad (Dinges and Powell, 1985; Honn et al., 2015). Participants rest their thumbs within a standard distance (a few millimeters) from the screen and tap immediately when red numbers appear in a centered box. Participants must use their dominant hand for accuracy. Less time (in seconds) to complete the task indicates a vigilant response.

The protocol also comprises cognitive assessments utilized in clinical practice, including subtests from the Wechsler Intelligence Scale for Children Fifth Edition (WISC-V) for participants ≤ 16 years of age and the Wechsler Adult Intelligence Scale Fourth Edition (WAIS-IV) for participants ≥17 years of age (Wechsler, 1949, 2008). The WISC-V is designed for ages 6–16 years and 11 months, while the WAIS-IV is designed for ages 16–90 years and 11 month. Hence, at 16 years of age, either assessment is appropriate, but for those 17 years and older, only the WAIS-IV is appropriate. Hence, we elected to have a cutoff age of 17 years old. This decision was justified by our goal of establishing a consistent protocol, and with no intention of using the measures clinically. The subtests administered include those required to obtain a Processing Speed Index (Coding and Symbol Search subtests) (Kramer et al., 2023). Participants are given the standard instructions and allotted 120 s (2 min) to complete each task individually. Raw scores are scaled for age during analysis. Participants also receive age-appropriate portions of the NIH Toolbox Cognitive Battery (Gershon et al., 2013; Weintraub et al., 2013) including the Dimensional Change Card Sort, Flanker Inhibitory Control and Attention, List Sorting Working Memory, Oral Reading Recognition, Pattern Comparison Processing Speed, Picture Sequence Memory, and Picture Vocabulary. Age standardized scores on these measures assess crystallized intelligence (stored knowledge) as well as executive functioning and other measures of fluid intelligence (e.g., problem solving, attentional control, processing speed, and memory).

#### 2.1.5 Emotion regulation

Caregivers complete the Behaviors Assessment System for Children Third Edition- Parent Rating Scales (BASC-3 PRS) (Reynolds and Kamphaus, 2015) to obtain their impressions of the participant's mood and behavior at home and in the participants' communities. Reports derived from these ratings provide information on internalizing and externalizing problems, adaptive skills, and emotional self-control.

### 2.2 Study design

The study is a single overnight cross-sectional study in the context of a pediatric hospital. Participants fill in a 7-day sleep diary prior to coming in for the sleep study. When they arrive at the hospital, participants are led to research space for consent and cognitive measures. Following completion of the cognitive assessments, participants are formally admitted to the hospital for the PSG and are then brought to the Sleep Center for the night. The next morning, participants are led from the Sleep Center back to the research office space to complete all other assessments. Participants receive payment and are accompanied as they transition between the research space and Sleep Center. Visits are completed in approximately 12 h.

### 2.3 Workflow

The protocol bridges between clinical and research assessments and incorporates expertise from different disciplines, including research laboratory personnel (clinical research coordinators and principal investigators), members of the Sleep Center team (sleep technicians, pulmonologists, administrators), hospital admissions employees (during the check-in process), and the security team. The workflow described below ensures proper communication between team members and an established timeline for collaboration with the clinical Sleep Center.

#### 2.3.1 Recruitment

If a family is eligible and interested in participating (confirmed through a phone call with our eligibility screeners), research coordinators contact the Sleep Medicine provider in the study team to enter a sleep study referral into the participant's medical record. This notifies the Sleep Center's scheduling team that we need to reserve a room/bed for research purposes. The referral will come with notes for the sleep technician, both informing them of the proper EEG montage for our study and the lack of need for any blood testing procedures during the visit (which is typically a standard of care for a clinical sleep study).

#### 2.3.2 Scheduling

Once the scheduling team members receives the referral from the Sleep Medicine provider, coordinators contact staff members involved in scheduling the visit as well as the caregiver. This procedure facilitates efficient communication between all parties and limits multiple touch points that could add to burden on the family and Sleep Center.

#### 2.3.3 Study visit–evening

Coordinators communicate arrival procedures with the family prior to the study day. This includes a form with reminders for the study night (e.g., arrival times) as well as a map of the hospital indicating where to meet their coordinator. Once the participant arrives alongside their caregiver, the coordinator meets them at the mentioned location and brings them to the research-dedicated space to sign informed consent documents as well as complete the immediate training sessions of the memory tasks and self-report documents. Once the family finishes these tasks and documents, the research study coordinator escorts them to the Admissions Office for formal admission to the hospital, and then to the Sleep Center. The coordinator confirms hookup procedures with the sleep technician and then leaves the family for the rest of the night.

#### 2.3.4 Study visit–morning

The following morning, at the Sleep Center, the coordinator returns after the sleep technician removes all PSG equipment and leads the family back to the research space to complete the 2nd day of memory tasks and cognitive measures. Once all measures are completed, the family receives compensation for the visit and departs the medical center.

#### 2.3.5 Post visit

To receive the PSG data, the coordinator requests the PSG data from the Sleep Center. This includes: the raw EEG files, the sleep scoring output, annotations, and the sleep interpretation report. The latter report summarizes information about the participant's sleep architecture, respiratory events, limb activity, cardiac activity, and, if applicable, a sleep disorder diagnosis.

### 2.4 Inclusion/exclusion criteria

The goal of including typically developing youth informs our inclusion/exclusion criteria. The study will include children and adolescents aged 8–19 years old who are fluent in English, as most of the measures we administer, as well as the consent/assent process, are in English. We include participants who are at least eight years old to ensure it is feasible for them, developmentally, to complete our memory tasks. We include participants up to19 years old as we aim to acquire data on participants across a broad range of cortical development and the Sleep Center in our pediatric hospital often assesses patients up to that age. Exclusionary criteria include a history of a sleep study for medical reasons, a neurodevelopmental disorder or intellectual disability (presence of an Individualized Educational Plan or Section 504 plan), a chronic illness effecting brain system development, severe mental illness, a sensory/motor impairment, active enrollment in a research study that affects participation in this study, and any medication known to significantly alter sleep brain oscillations (e.g., selective serotonin reuptake inhibitors) Exclusion parameters also include answering “yes” to “snoring more than half of the time” or “always snore” in the Pediatric Sleep Questionnaire (PSQ) (Chervin et al., 2000), indicating the possibility of sleep disordered breathing.

### 2.5 Recruitment and scheduling

We utilize multiple recruitment strategies to ensure our sample is representative of healthy typically developing children and adolescents residing in Franklin County, Ohio. Recruitment efforts leverage the following methodologies: (1) digital signage within our hospital; (2) monthly mass emails to hospital employees; (3) social media advertisements (via our hospital's marketing department); (4) paper fliers within the Franklin County community; (5) posts on a website supported by The Ohio State University Clinical and Translational Science Institute that advertises research studies to potentially interested participants residing in the surrounding area; (6) advertising the study to a list of contacts who had participated in a previous research study within our hospital and consented to be contacted about future studies; (7) partnership with a primary care school based health program (which works with providers in the local school system); and (8) encouraging participants who completed the study to share information about the study with family and friends.

#### 2.5.1 Digital signage

We utilize digital signage within the hospital to recruit participants. Digital boards throughout the hospital display our flyer on an image carousel that plays for 2 weeks out of the month. Participants can contact us using the information on the boards. Note that the duration of slides on the carousel can affect the amount of detail families can gather about a study.

#### 2.5.2 Mass email to employees

Our hospital offers the opportunity for employees to sign up to a mailing list that advertises opportunities to participate in research studies. Our protocol involves sending an email to said mailing list, informing employees of a study and of the lead coordinator's contact information. Interested participants are invited to contact the coordinator by email or phone to determine if the family is eligible for participation.

#### 2.5.3 Social media advertisements

Advertisements on social media platforms are curated through the marketing department of our hospital and target anyone with relation to a child/adolescent in the age range of our target participants. Building on existing policies of outreach of our hospital, we found that there was easy access to advertising in two platforms (Instagram and Facebook). Advertisements are set to run for two consecutive weeks each month and include information from our flyer and a hyperlink connecting to a REDCap (Harris et al., 2009, 2019) survey. This survey includes questions about interest in study participation and a digital version of the PSQ. Study coordinators contact families that indicate an interest in participating to determine if their child or adolescent meets eligibility criteria. Limitations of the social media ads include their expense and lack of outreach to families without internet/social media accounts.

#### 2.5.4 Community fliers

As an additional means for recruiting participants, our lab posts fliers that include study details and a QR code that links participants to a REDCap survey from the social media ads. Fliers are placed at local coffee shops, public libraries, community centers, and Starbucks locations that have community boards.

#### 2.5.5 Online research hub

We also utilize StudySearch.com as a method of recruitment. This website is a tool for recruiting families affiliated with the Ohio State University community who have expressed an interest in participating in research. The website lists active studies classified by research topic, showing for each study the eligibility criteria as well as a study summary and the study coordinator's contact information.

#### 2.5.6 Existing databases of potentially interested community families

An additional recruitment method is to collaborate with a research colleague who has an ongoing study with a built-in repository of participants who consented to being contacted in the future about other research studies. Leveraging this repository, we contact control participants who have previously been willing to participate in research and may be likely to participate again.

#### 2.5.7 Primary care-based school outreach program

A final recruitment strategy is to partner with NCH Primary Care School Based Health, a program that involves students from local schools who are served by physicians affiliated with our hospital in research. Our study team provides fliers to the physicians and explain the study in detail to help them inform potential participants within schools. Interested families can then contact the study coordinator directly.

### 2.6 Ensuring data quality

#### 2.6.1 Recruitment troubleshooting

A goal of our study is to ensure we are recruiting a sample that is representative of the county where our hospital resides. To achieve a sample distribution of participant age that corresponds with study objectives, we suggest that studies implementing our protocol monitor the age distribution of current participants every few months and introduce time in which advertisement postings selectively target recruitment to specific ages. We also design our advertisement fliers to include images that are broadly representative of the targeted sample. To track recruitment success, we suggest that studies track the method used to recruit each participant, and periodically evaluate which strategies are most efficient. Less efficient strategies may be worth implementing if they allow for recruitment of underrepresented groups (for example, fliers posted in a primary care office might not be highly effective but might help increase recruitment in targeted zip codes).

#### 2.6.2 Data curation

Participants whose PSG reveals incidental findings after clinical review by the pediatric sleep medicine expert in the study team (Dr. Kalra) will not be included in the final analysis to test the hypotheses in our specific aims. In analyzing data from sleep diaries, we plan to exclude from final analyses data from participants for whom fewer than 4 days of the diaries were reported. For sleep diaries that report at least 4 days but not seven, we will treat the missing data through imputation, specifically via multivariate imputation by chained equations (Van Buuren and Groothuis-Oudshoorn, 2011; White et al., 2011). Memory performance in the CGT and TDT in the morning after PSG might be affected by how rested participants will feel after an in-lab PSG. Performance in the PVT, which is administered closest to waking, will serve as a metric of restfulness. Participants with very poor PVT score (average response time > 300 ms, see (Basner et al., 2020), if any, will be excluded from analyses of overnight changes in memory performance.

### 2.7 Data analysis plan

While this research protocol provides rich multi-modal information on many sleep and cognitive domains, our analysis plan is designed to address the two study aims: (1) establish age-dependent changes in the space-time organization of sleep slow oscillations on the electrode manifold; and (2) explore if metrics that quantify the space-time organization of SOs are related to sleep-dependent changes in episodic memory in youth. The space-time organization of sleep SOs is derived from the co-occurrence of events at a short delay (Malerba et al., 2019; Niknazar et al., 2022; Alipour et al., 2025; Snedden et al., 2026). We will use the percentage of Global SOs found in non-rapid eye movement (NREM) stages 2 and 3 sleep (labeled %G) as the metric of space-time organization of SO for both aims (primary outcome variable). We will use the overnight change in performance (ratio of performance after sleep over performance before sleep, expressed as percentage) for the CGT (labeled dP_CGT_) as the metric for change in overnight memory performance (secondary outcome variable). Given that the spatial presentation of SOs tends to track the development of the frontal lobe (Kurth et al., 2010), we hypothesize that we will find a significant negative correlation between age and %G in typically developing youth in aim 1. For aim 2, we hypothesize that we will find a significant positive correlation between %G and dP_CGT_. This hypothesis is based on literature supporting a role for SOs and other sleep rhythms of NREM sleep in consolidation of episodic memory (Marshall et al., 2006; Kurdziel et al., 2013; Mednick et al., 2013), while perceptual memory seems to be related to both N3 and REM sleep activity (Karni et al., 1994; Stickgold et al., 2000; Mednick et al., 2003).

#### 2.7.1 Analyses of sleep EEG to determine the fraction of global SOs (%G)

Sleep EEG data acquired during polysomnography will be analyzed with custom scripts in Matlab (TheMathWorks, Nantucket, MA, United States) to achieve identification of sleep SOs. This will be achieved following established procedures deployed in examining multiple sleep datasets (Simon et al., 2021). Following detection of SO events, their space-time organization will be determined based on co-occurrence of the SO trough across multiple electrodes within a short time delay (400 ms). This methodology has been leveraged in other datasets of sleep EEG (Malerba et al., 2019; Niknazar et al., 2022; Alipour et al., 2025), and yields a classification of each SO event as Global, Local or Frontal. Global SOs are widespread events, with troughs found at most electrodes within the short delay, and, in comparison to Local and Frontal SOs, have been shown to have largest amplitude and most effective coordination with spindles (Malerba et al., 2019). For each participant, the total count of Global SO during stage 2 and 3 NREM sleep (totG) will be divided by the total number of SOs during stage 2 and 3 NREM (totSO), to compute %G = 100^*^totG/totSO. This metric, derived from the sleep EEG and representative of the degree of coordination of SO events across the electrode manifold, will be used as outcome measure for statistical analyses.

#### 2.7.2 Power analysis

We plan to collect data on 95 participants, for a final sample size of 90. Power analyses were conducted in RStudio (Posit Software, 2025). There is no exact preliminary data to establish an expected correlation between age and %G in youth 8–19 years old, so our power analysis is estimate-based. In an unpublished preliminary analysis of data on the relation between age and %G in a dataset of single night sleep in young adults (18–34), we found a negative correlation of −0.37 (Pearson's, *p* < 0.01; data from Dr. Sara Mednick at University of California Irvine). Based on this data, we estimate that a sample of 85 individuals could provide at least 80% power at a significance threshold of 0.05 to detect a Spearman's correlation of 0.3 or greater between age and the outcome (%G). Similarly, there are no exact data that can function as preliminary data for the relation between %G and overnight changes in score in the CGT. Preliminary data from (Kurdziel et al. 2013) indicated a positive correlation of 0.647 (Pearson's. *p* < 0.05) between spindle density and change in memory performance in the CGT in a sample of 19 participants. Based on this data, we can estimate that a sample of 22 individuals could provide at least 80% power at a significance threshold of 0.05 to detect a Spearman's correlation of 0.55 or greater between %G and dP_CGT_. The final participant recruitment target of 95 is derived by considering a potential attrition of about 12%, to account for partial completion of memory tasks and potential incidental findings in the PSG (see Section 2.6.2), and ensure a final sample size of 90.

#### 2.7.3 Statistical analysis plan

We will test all hypotheses with Spearman's correlations, with statistical significance at 0.05 threshold. Additional adjusted analysis, using partial correlation or linear regression, will be conducted to control for potential confounders, such as pubertal status, sex, and other demographic factors, as well as to investigate moderators of associations of age with %G in aim 1 (expected to be monotonic) and of %G with dP_CGT_ in aim 2.

## 3 Discussion

This article describes a protocol for acquiring sleep and cognition in typically developing youth who are evaluated as research participants at a children's hospital. Our multidomain protocol includes metrics of sleep, cognition, behaviors, and socio-demographic measures. Sleep is assessed both at home (with sleep diaries) and in laboratory, with a single overnight sleep study in a large Midwestern children's hospital. This allows consideration for subjective and objective measures of sleep in the same participants, providing careful characterization of sleep physiology (via PSG) that can be contextualized with sleep patterns and daytime behaviors that can impact sleep (Simon et al., 2025). Similarly, our protocol will assess cognition using both established psychometric tools commonly used in the clinic and laboratory-based memory tasks that have been shown to be sensitive to overnight sleep neurophysiology. Again, this allows for convergence of perspectives on studying the relation between sleep and cognition that are derived from clinical research and basic science, thus establishing a truly translational protocol.

While the protocol we present here is focused on typically developing youth, this experimental approach can function as a blueprint to enable studies of sleep (high density EEG) and cognition in both clinical populations and typically developing controls, including repeated assessment to examine longitudinal change within individuals (Simon and Duggan, 2026). When extending the presented approach to a study including a pediatric population, the choice of which cognitive assessments to use will depend on the research goal and population of interest. Our protocol was designed to fit the needs of a study involving effects of sleep on tests of memory performance before and after a nighttime PSG. For this study, we choose memory tasks that are well suited to the study of changes in memory performance across sleep episodes, as evinced by the multiple studies of sleep and cognition in which they have been deployed. Appropriateness of a task for this specific type of study (changes in performance across sleep episodes) is established based on the task being set up to minimize/eliminate influence of learning across multiple testing, adjusting thresholds for task training to allow for measurable effects of sleep (i.e., avoid over-training), and appropriateness of the task to the skill level of the population. Thus, when adapting the protocol for clinical populations, we advise researchers to conduct pilot evaluation of the fit of memory tasks procedures to the skills and need of the pediatric population of interest. Depending on study aims and consideration of participant burden, other measures may also be selected to evaluate behavior and mood, pubertal status, and cognition. Similarly, depending on the goal of the study, researchers adapting this protocol might consider including actigraphy at home to coincide with the days in which the sleep diary is collected.

One limitation of this protocol is that some clinical sleep centers have a common early wake time (e.g., 5 a.m.) due to sleep technician work schedules and early school start times. As early wake times can result in reduced acquisition of morning REM sleep, investigators may want to negotiate an alternative wake time for their participants. However, as early school times are common and known to impact sleep duration in youth (Carskadon et al., 1998), use of early wake times might be appropriate when exploring potential relations between sleep metrics derived from PSG and cognitive measures (e.g., executive functioning, or cognitive speed), at least for those participants that report early wake times in their sleep diaries. A further limitation of the protocol is the lack of an adaptation night. Researchers may elect to add a preparatory first night to their protocol if piloting suggests that youth have difficulties adapting to the sleep environment. Of note, previous research indicates that first night effects (FNE) reduce time in REM sleep by 2%−5%, but have a limited impact on N2 and N3 sleep (Lorenzo and Barbanoj, 2002; Ding et al., 2022; Wick et al., 2024), suggesting that our primary outcome (fraction of Global SOs in N2 and N3 sleep) may not be significantly impacted by FNE. Similarly, SO properties like amplitude and density have shown high test-retest reliability in an analysis of four sleep nights collected 1 week apart (unpublished data, 13 adults, intraclass correlation across four nights is 0.76 with confidence interval 0.54–0.90 for SO amplitude in SWS, data from Dr. Mednick's laboratory at UC Irvine). This suggests that SO space-time properties are also going to be highly stable across multiple nights, supporting appropriateness of a single-night measurement. Considering standard PSG metrics, FNEs have been seen in wake after sleep onset, sleep onset latency, and total sleep time. Thus, when reporting PSG findings from this protocol, it will be appropriate to discuss them in the context of this literature. An additional limitation of the study is the requirement for English fluency, which precludes assessment of the broader population of potential participants. Extension of the research protocol to other participants with primary languages other than English would provide an opportunity for broader application and generalization of study findings. Finally, this protocol uses a cross-sectional design to examine age-related differences. Studies of longitudinal change in the association of sleep and cognition, or of the effects of an intervention, would require repeated assessments across multiple nights.
