# Supplementary material for: Assessing sleep, cognition and overnight memory performance in neurotypically developing youth in a children's hospital
Source: Front Sleep. 2026 Jun 4;5:1771860. doi: 10.3389/frsle.2026.1771860 (PMC13275221; doi:10.3389/frsle.2026.1771860)
Supplement: Supplementary file 1 [file Data_Sheet_1.pdf]

Supplementary Information:
Assessing sleep, cognition and overnight memory performance in neurotypically developing youth in a children's hospital

Gabrielle N. Deutsch, Emmet W. Klein, Chelsea E. Cadle, Katharine C. Simon, H. Gerry Taylor, Maninder Kalra, Paola Malerba

Table S1 – Study measures

Assessment/ Measure	Administration Setting & Context	Measure Construct	Rationale	Reference
PDS	A 5-item self-report questionnaire regarding pubertal status.	Pubertal status	Used to better account for differences in cognitive and pubertal development for adolescents. Cronbach alpha between .91-.96	(Petersen et al., 1988; Koopman-Verhoeff et al., 2020)
PSG in a clinical laboratory	Overnight sleep study. This includes EEG, EMG, EOG, ECG, and respiration measurements.	Objective overnight sleep measurement	Gold standard	(Grigg-Damberger, 2012)
ESS-CHAD	8-item self-report questionnaire filled out by the participant. A score ≥10 is indicative of abnormal daytime sleepiness.	Daytime sleepiness	Validated in 12–18-year-olds with a Cronbach alpha score of 0.73	(Johns, 2015; Janssen et al., 2017)

ESS	8-item self-report questionnaire filled out by participant 18 years of age and older. A score ≥10 is indicative of abnormal daytime sleepiness.	Daytime sleepiness	Validated in participants 18 years of age and older with a Cronbach alpha score of 0.88	(Johns, 1991; Doneh, 2015)
Sleep Diary	A sleep diary developed for teenagers leveraging the National Sleep Foundation Diary. Participants log morning and evening behaviors for 7 consecutive days.	At home sleep	Used to establish at-home sleep across a 7-day period	(Carney et al., 2012; Knutson et al., 2017)
CGT	Episodic memory task. Participants complete encoding and immediate testing before PSG hookup and delayed testing the following morning.	Episodic memory	The CGT was chosen as an engaging visuospatial episodic memory task with known sleep dependence in youth.	(Kurdziel et al., 2013; Sonni and Spencer, 2015)
TDT	A computer-based visual textured perceptual memory task. Participants complete training	Procedural memory	Several studies have supported the sleep-dependent enhancement of	(Karni and Sagi, 1991; Ahmadi et al., 2018)

	and immediate testing before PSG hookup and delayed testing the following morning.		a foreground–background discrimination task (i.e., Visual texture discrimination).	
PVT	5-minute sustained attention and reaction time task completed on an iPad immediately following wake up from PSG.	Vigilance	Assess subjects' level of alertness and attention after PSG.	(Basner and Dinges, 2012; Khitrov et al., 2014)
WISC-V/ WAIS-IV	Intelligence test that measures intellectual ability. Participants complete the processing speed index which includes the coding and symbol search subtests. Participants younger than 16 years old will complete the WISC while 17+ will complete the WAIS task the morning following PSG.	Processing speed; Working Memory; Cognitive Speed	Standard cognitive assessment utilized in clinical practice	(Wechsler, 1949; Wechsler, 2008)

NIH Toolbox Cognitive Battery	A cognitive battery consisting of 7 subtests related to executive functioning and memory. These tests are split up into Fluid, Crystallized, and Total Cognition scores.	Executive functioning and memory	A standard assessment tool commonly deployed in clinical psychology practice.	(Weintraub et al., 2014)
BASC-3 PRS	Parent-report questionnaire regarding behaviors in the community and at home. Reports derived from the measure include emotion regulation, internalizing problems, adaptability, etc.	Adaptability and behavioral problems.	Coefficient alpha is .94 for children 8-11 years old, and .97 for adolescent 12-18 years old. Norms were developed on large, demographically representative samples that enhance the interpretability of scores.	(Reynolds, 2010)

Note. Pediatric Sleep Questionnaire (PSQ), Epworth Sleepiness Scale for Children and Adolescents (ESS-CHAD), Polysomnography (PSG), Card Grid Task (CGT), Texture Discrimination Task (TDT), Behavioral Assessment System for Children Version 3 Parent Rating Scale (BASC-3 PRS), Psychomotor Vigilance Task (PVT), Weschler intelligence Scale for Children 5th Edition (WISC-V), Weschler Adult Intelligence Scale 4th Edition (WAIS-IV), Pubertal Development Scale (PDS).

Figure S1

Sleep Diary

Complete in Morning							
Day of the Week:	Day 1 ____	Day 2 ____	Day 3 ____	Day 4 ____	Day 5 ____	Day 6 ____	Day 7 ____
I got into bed last night at…	PM/AM	PM/AM	PM/AM	PM/AM	PM/AM	AM/PM	AM/PM
I fell asleep at…	AM/PM	AM/PM	AM/PM	AM/PM	AM/PM	AM/PM	AM/PM
I woke up during the night…							
# of times							
# of minutes							
I woke up this morning at…	AM/PM	AM/PM	AM/PM	AM/PM	AM/PM	AM/PM	AM/PM
I got out of bed at…	AM/PM	AM/PM	AM/PM	AM/PM	AM/PM	AM/PM	AM/PM
When I woke up for the day, I felt…							
Rested<br>Somewhat rested<br>Tired	☐<br>☐<br>☐	☐<br>☐<br>☐	☐<br>☐<br>☐	☐<br>☐<br>☐	☐<br>☐<br>☐	☐<br>☐<br>☐	☐<br>☐<br>☐
Notes: Record any other factors that may have affected your sleep							

Complete in Evening							
Day of the Week:	Day 1 ____	Day 2 ____	Day 3 ____	Day 4 ____	Day 5 ____	Day 6 ____	Day 7 ____
I consumed caffeinated items in the: (M)orning, (A)fternoon, (E)vening, (N/A) (e.g. soda, tea, coffee, energy drinks, chocolate)							
M/A/E/NA<br><br>How much?							

**I exercised at least 20 minutes in the: (M)orning, (A)fternoon, (E)vening, (N/A)**							
**M/A/E/NA**							
**I took these medications today:**							
**Took a nap?** **(Circle one)**	**Yes** **No**	**Yes** **No**	**Yes** **No**	**Yes** **No**	**Yes** **No**	**Yes** **No**	**Yes** **No**
**If yes. For how long**							
**Throughout the day, my mood was…** Very pleasant (**VP**), Pleasant (**P**), Unpleasant (**UP**), Very unpleasant (**VUP**)							
VP/P/UP/VUP							
**In the hour before going to sleep, my bedtime routine included: list of activities including reading a book, taking a bath, doing relaxation exercises, etc.**							
**In the hour before going to sleep, I used electronics (e.g. cell phone iPad/tablet, computer, TV video games)**							

This 7-day sleep diary was derived from the National Sleep Foundation sleep diary and has been adjusted for teenagers.

References

Ahmadi, M., McDevitt, E.A., Silver, M.A., and Mednick, S.C. (2018). Perceptual learning induces changes in early and late visual evoked potentials. *Vision Res* 152, 101-109. doi: 10.1016/j.visres.2017.08.008.

Basner, M., and Dinges, D.F. (2012). An adaptive-duration version of the PVT accurately tracks changes in psychomotor vigilance induced by sleep restriction. *Sleep* 35(2), 193-202. doi: 10.5665/sleep.1620.

Carney, C.E., Buysse, D.J., Ancoli-Israel, S., Edinger, J.D., Krystal, A.D., Lichstein, K.L., and Morin, C.M. (2012). The consensus sleep diary: standardizing prospective sleep self-monitoring. *Sleep* 35(2), 287-302. doi: 10.5665/sleep.1642.

Doneh, B. (2015). Epworth Sleepiness Scale. *Occupational Medicine* 65(6), 508-508. doi: 10.1093/occmed/kqv042.

Grigg-Damberger, M.M. (2012). The AASM Scoring Manual four years later. *J Clin Sleep Med* 8(3), 323-332. doi: 10.5664/jcsm.1928.

Janssen, K.C., Phillipson, S., O'Connor, J., and Johns, M.W. (2017). Validation of the Epworth Sleepiness Scale for Children and Adolescents using Rasch analysis. *Sleep Med* 33, 30-35. doi: 10.1016/j.sleep.2017.01.014.

Johns, M. (2015). The assessment of sleepiness in children and adolescents. *Sleep Biol Rhythms* 13(Suppl 1), 97.

Johns, M.W. (1991). A New Method for Measuring Daytime Sleepiness: The Epworth Sleepiness Scale. *Sleep* 14(6), 540-545. doi: 10.1093/sleep/14.6.540.

Karni, A., and Sagi, D. (1991). Where practice makes perfect in texture discrimination: evidence for primary visual cortex plasticity. *Proc Natl Acad Sci U S A* 88(11), 4966-4970. doi: 10.1073/pnas.88.11.4966.

Khitrov, M.Y., Laxminarayan, S., Thorsley, D., Ramakrishnan, S., Rajaraman, S., Wesensten, N.J., and Reifman, J. (2014). PC-PVT: a platform for psychomotor vigilance task testing, analysis, and prediction. *Behav Res Methods* 46(1), 140-147. doi: 10.3758/s13428-013-0339-9.

Knutson, K.L., Phelan, J., Paskow, M.J., Roach, A., Whiton, K., Langer, G., et al. (2017). The National Sleep Foundation's Sleep Health Index. *Sleep Health* 3(4), 234-240. doi: 10.1016/j.sleh.2017.05.011.

Koopman-Verhoeff, M.E., Gredvig-Ardito, C., Barker, D.H., Saletin, J.M., and Carskadon, M.A. (2020). Classifying Pubertal Development Using Child and Parent Report: Comparing the Pubertal Development Scales to Tanner Staging. *J Adolesc Health* 66(5), 597-602. doi: 10.1016/j.jadohealth.2019.11.308.

Kurdziel, L., Duclos, K., and Spencer, R.M.C. (2013). Sleep spindles in midday naps enhance learning in preschool children. *Proceedings of the National Academy of Sciences* 110(43), 17267-17272. doi: doi:10.1073/pnas.1306418110.

Petersen, A.C., Crockett, L., Richards, M., and Boxer, A. (1988). A self-report measure of pubertal status: Reliability, validity, and initial norms. *Journal of youth and adolescence* 17(2), 117-133.

Reynolds, C.R. (2010). Behavior assessment system for children. *The Corsini encyclopedia of psychology*, 1-2.

Sonni, A., and Spencer, R.M.C. (2015). Sleep protects memories from interference in older adults. *Neurobiology of Aging* 36(7), 2272-2281. doi: https://doi.org/10.1016/j.neurobiolaging.2015.03.010.

Wechsler, D. (1949). Wechsler intelligence scale for children.

Wechsler, D. (2008). Wechsler adult intelligence scale--(WAIS-IV)[Database record]. *Apa PsycTests* 10.

Weintraub, S., Dikmen, S.S., Heaton, R.K., Tulsky, D.S., Zelazo, P.D., Slotkin, J., et al. (2014). The Cognition Battery of the NIH Toolbox for Assessment of Neurological and Behavioral Function: Validation in an Adult Sample. *Journal of the International Neuropsychological Society* 20(6), 567-578. doi: 10.1017/S1355617714000320.
